# Supplementary material for: Extracellular Vesicles from Steatotic Hepatocytes Provoke Pro-Fibrotic Responses in Cultured Stellate Cells
Source: Biomolecules. 2022 May 13;12(5):698. doi: 10.3390/biom12050698 (PMC9138794; doi:10.3390/biom12050698)

Koenen et al., 2022 - Original western blot images

Figure 1E*

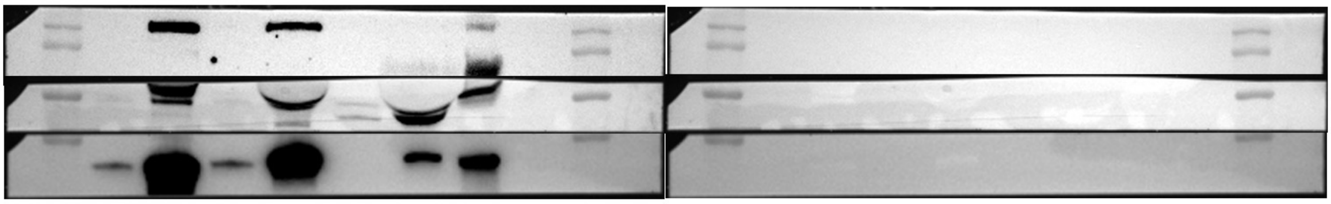

* Membrane was physically cut and developed separately by experimenter (left: ECL, right: light)

Figure 4F: actin

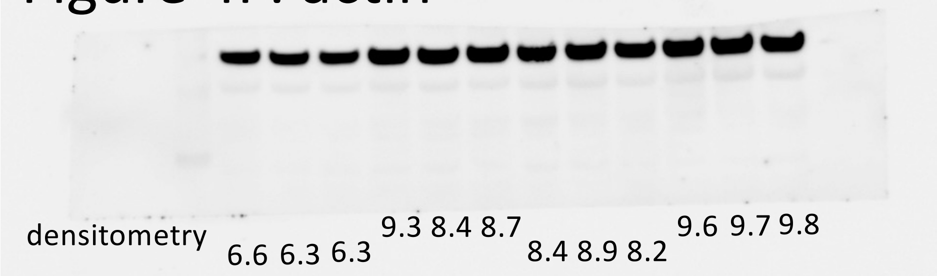

Figure 4F: GFAP

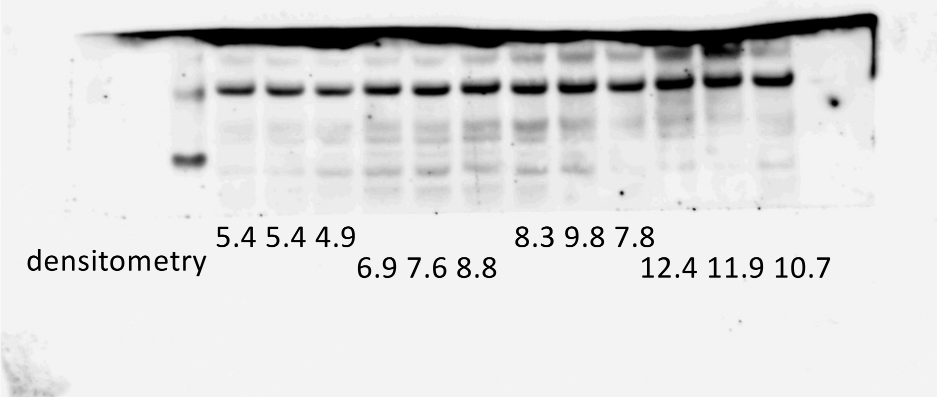

Figure 4G: vimentin and actin**

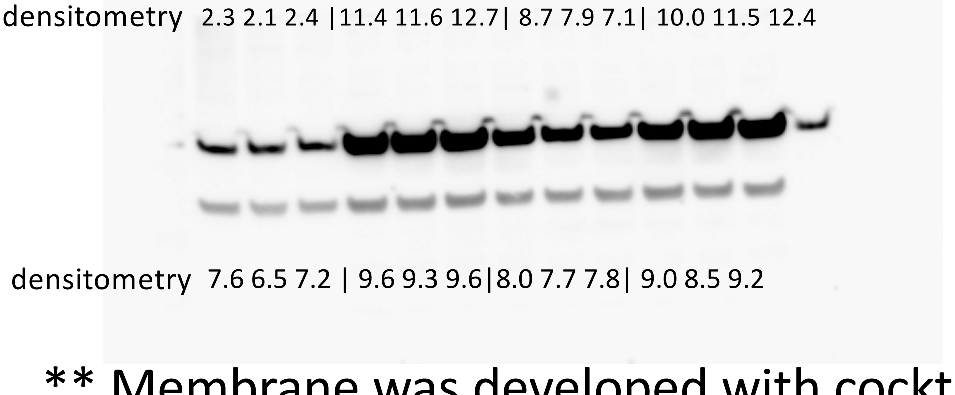

** Membrane was developed with cocktail of anti-vimentin and anti-actin (top signal: vimentin, lower signal: actin)

Koenen et al., 2022 - Replicate NTA traces

Figure 1A: NTA analysis of C-EV

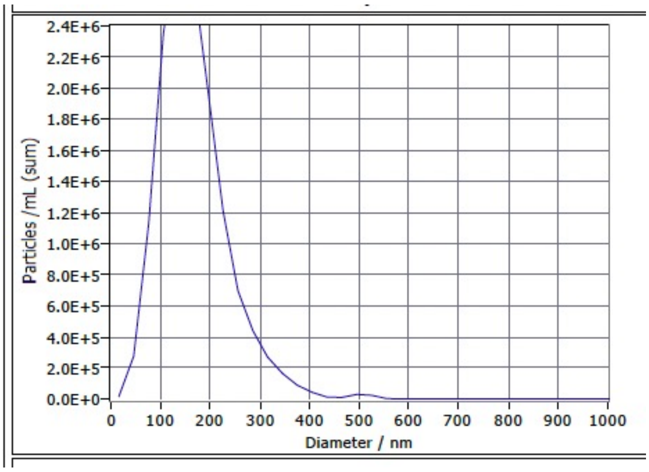

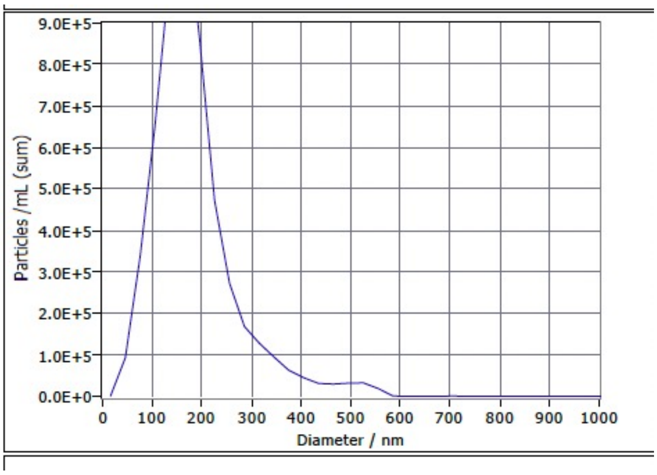

Figure 1A: NTA analysis of FA-EV

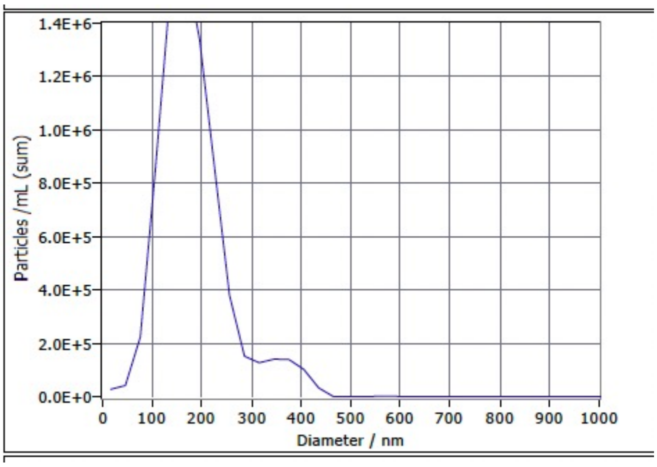

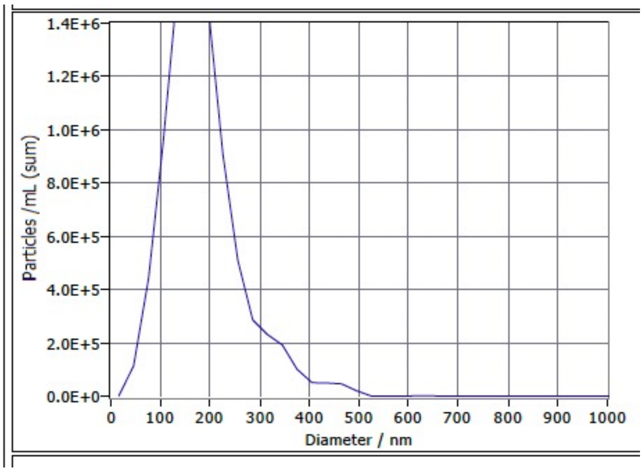

Koenen et al., 2022 - Replicate microscopy images

Figure 1B: Electron microscopy of C-EV

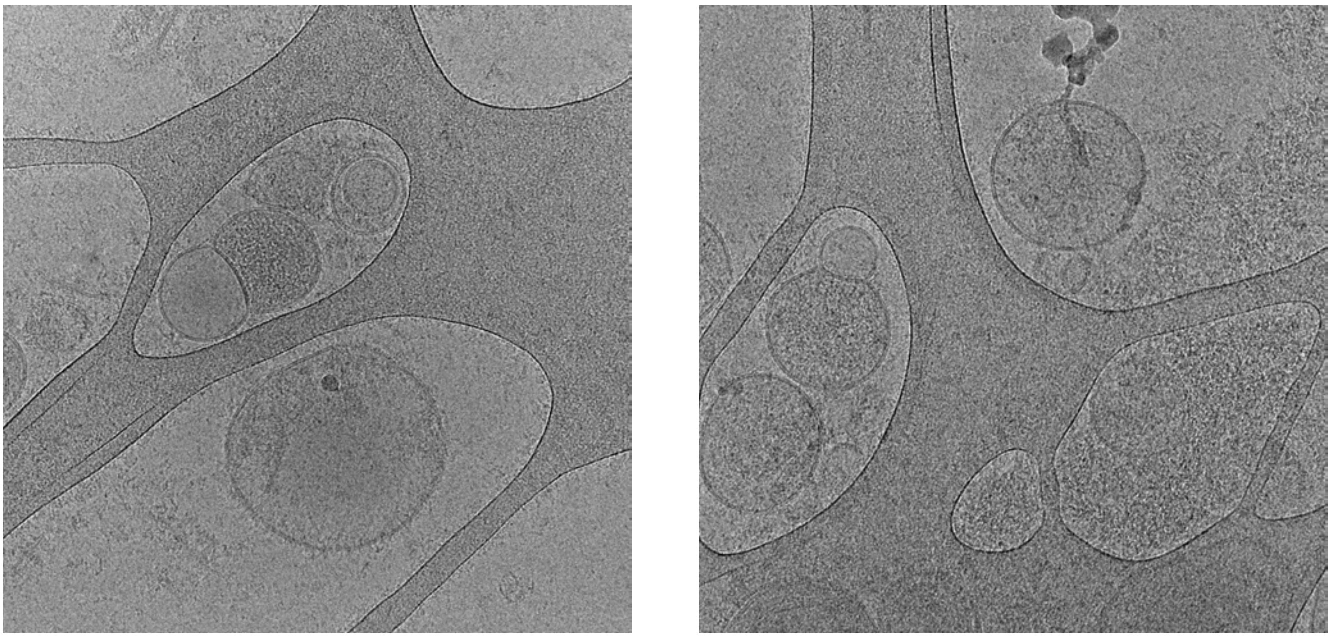

Figure 1B: Electron microscopy of FA-EV

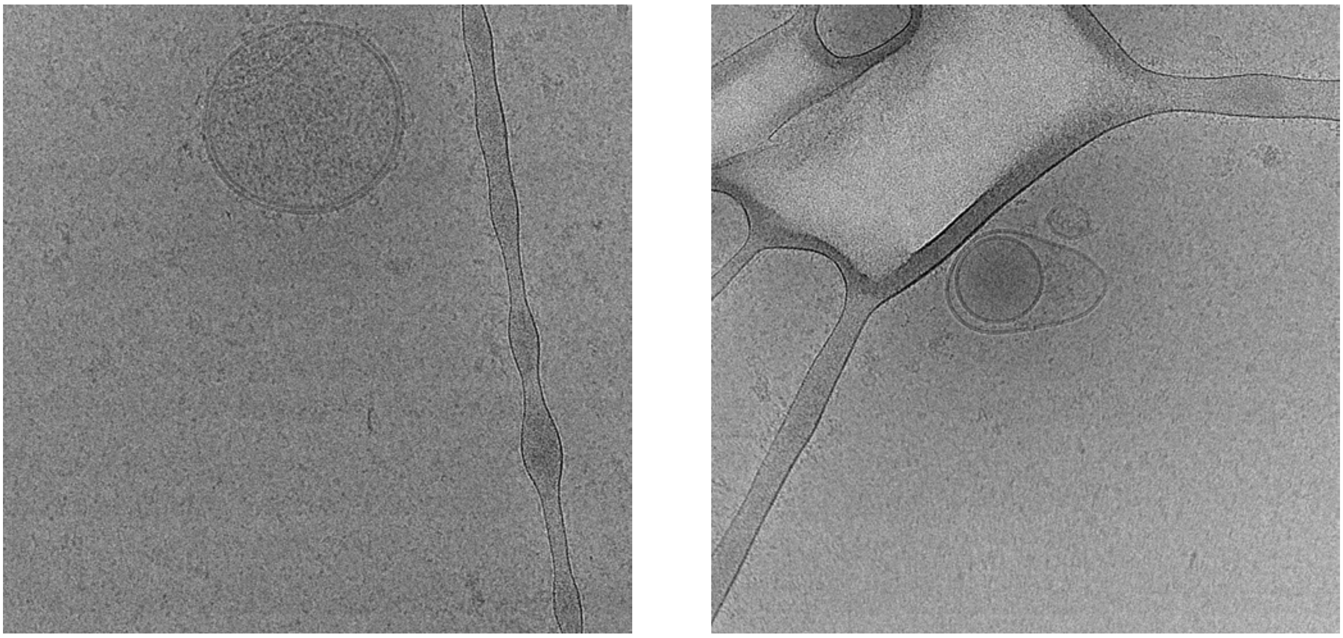

Koenen et al., 2022 - Replicate microscopy images

Figure 4A: actin-staining and DAPI

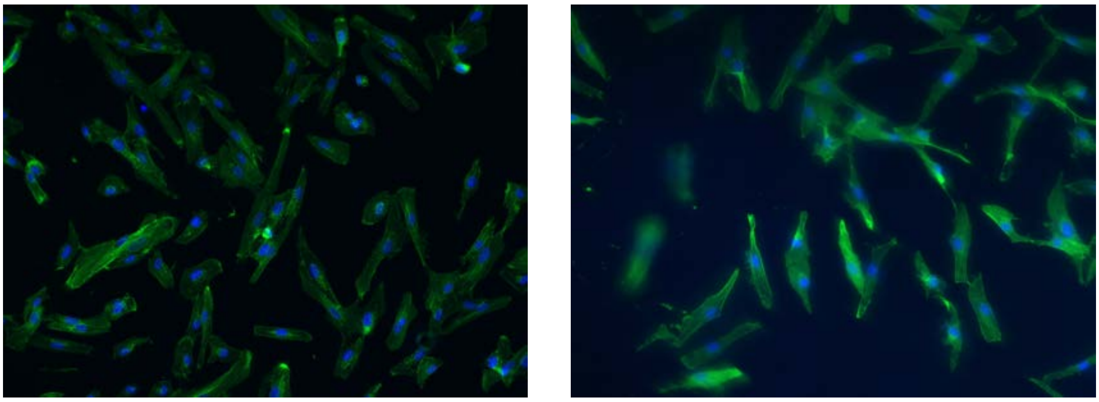

Figure 4B: actin-staining and DAPI

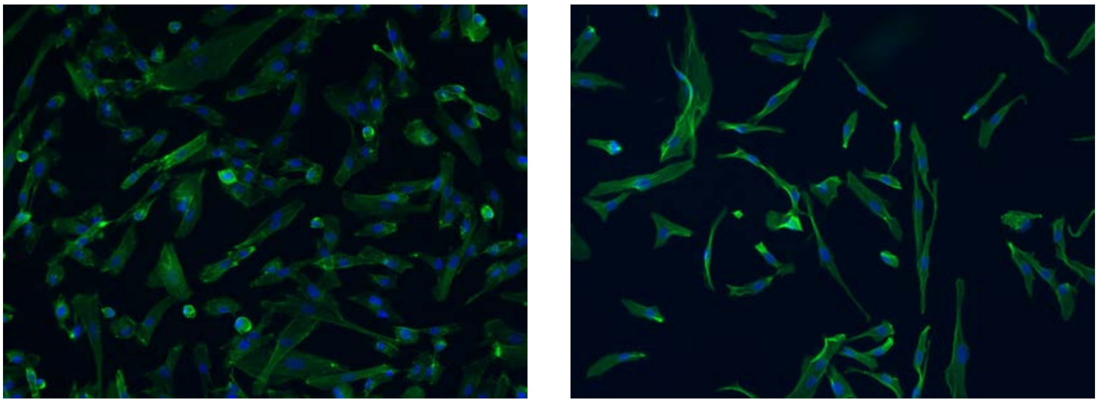

Figure 4C: actin-staining and DAPI

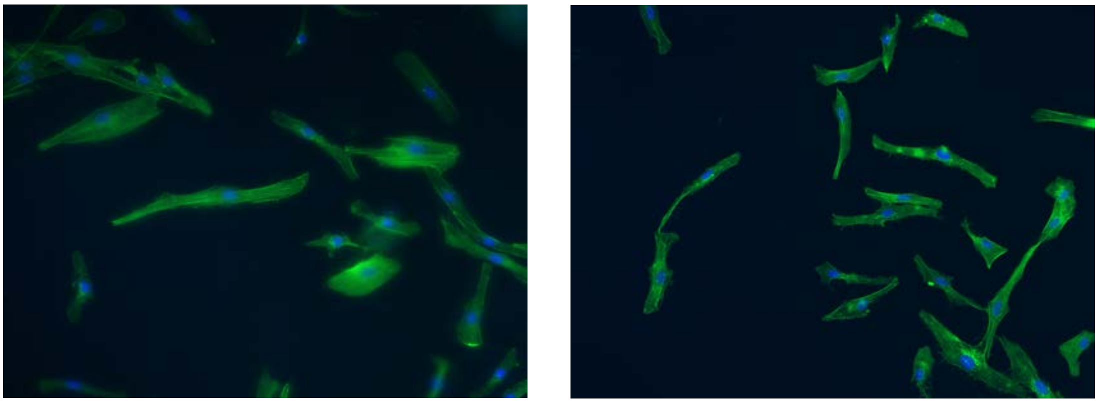

Koenen et al., 2022 - Replicate microscopy images

Figure 5A: Collagen, actin and DAPI

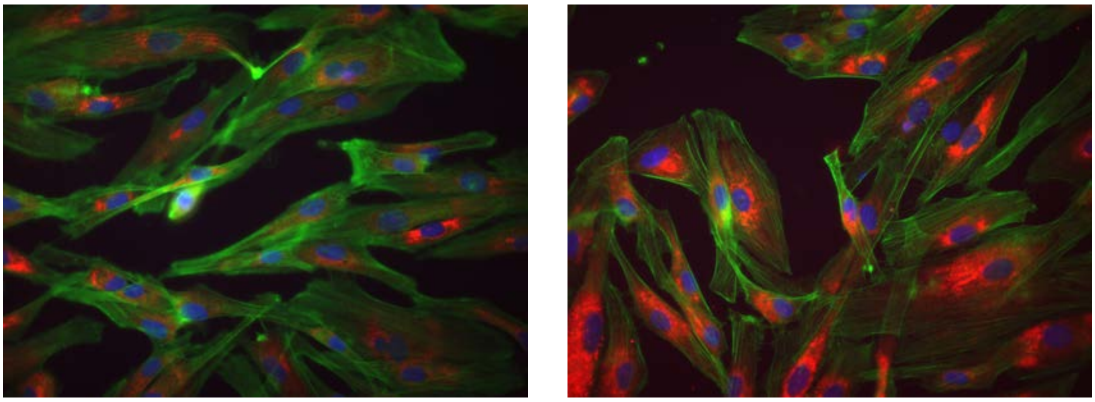

Figure 5B: Collagen, actin and DAPI

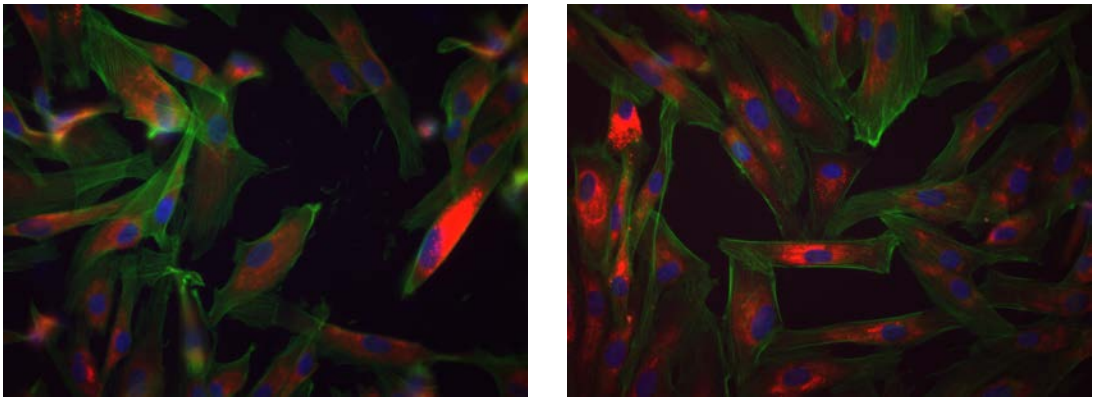

Figure 5C: Collagen, actin and DAPI

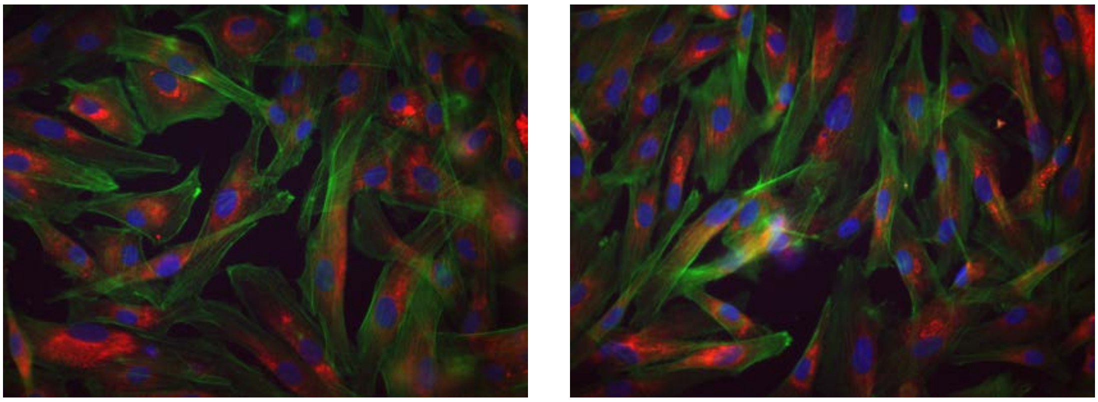

Koenen et al., 2022 - Original microscopy images

Figure A1: HepG2 Oil Red O

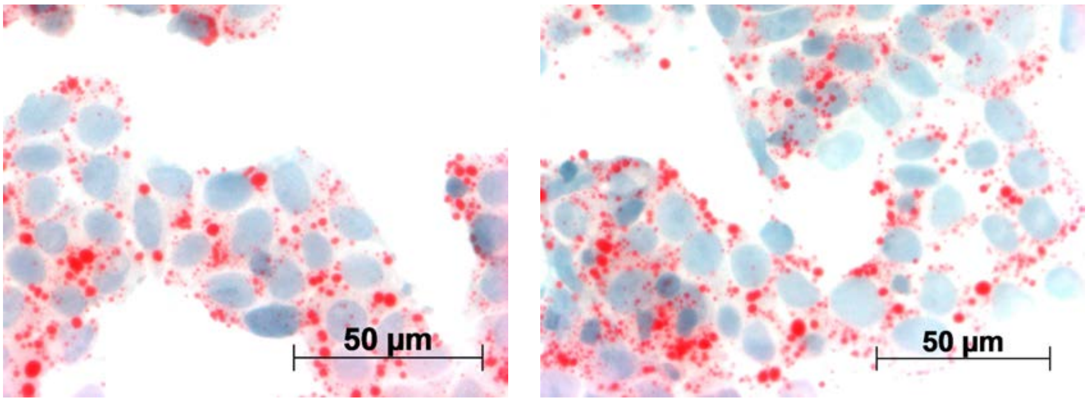

Figure A1: HepG2+FA Oil Red O

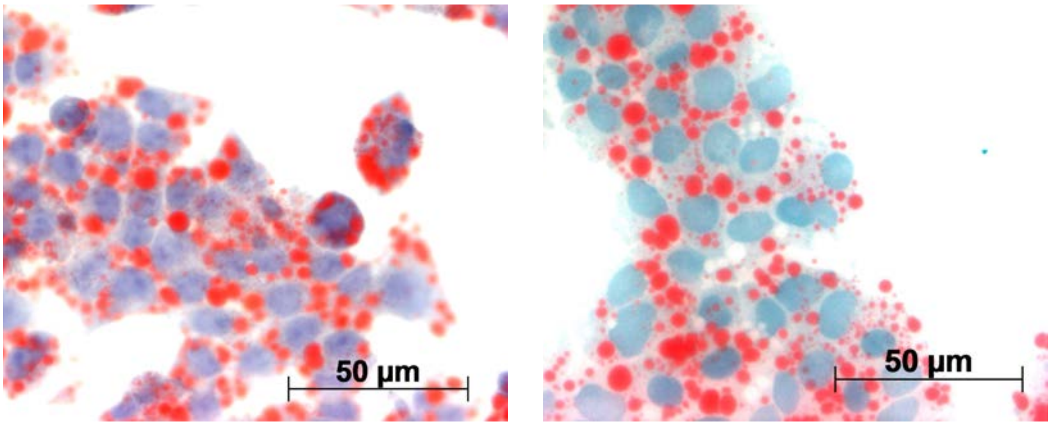

Supplement: Supplementary file 1 [file biomolecules-12-00698-s001.zip › biomolecules-1692225-supplementary.pdf]
